# Test feed development and methodological approaches allowing highly controlled dietary exposures to nano- and microparticulate contaminants in fish

**DOI:** 10.1016/j.mex.2020.101206

**Published:** 2020-12-30

**Authors:** Tobias Lammel

**Affiliations:** Department of Biological and Environmental Sciences, University of Gothenburg, Medicinaregatan 18 A, Box 463, 413 90 Göteborg, Sweden

**Keywords:** Test diet, Nanoparticle, Nanomaterial, Microplastic, Intestinal uptake, Bioaccumulation, Trophic transfer, Gut, Stickleback, Trout, Toxicity, Fate, Aquatic

## Abstract

There is increasing concern that particulate contaminants including manufactured nanomaterials and nano- and microplastics taken up and or accumulating in lower-trophic level aquatic organisms results in dietary exposure of fish feeding on these organisms. Controlled feeding studies can help advance our understanding of dietary uptake, bioaccumulation, and associated effects of (nano)particulate contaminants in fish, and also provide information about their likelihood to be transferred along the trophic chain and or to act as vector for other, surface-adsorbed pollutants. However, traditional approaches to prepare test feed for dietary exposure studies where commercial fish feed such as flakes, granules or pellets are soaked or spray-spiked with dissolved chemicals are not well suitable for (nano-)particulate contaminants. Microplastics, which often have sizes in the µm to mm range, and manufactured nanomaterials, in particular those which are soluble, such as metal/metal oxide nanoparticles, have to be *incorporated* into the feed to avoid their dissociation and or dissolution before the feed is ingested by the animal to avoid undesired waterborne exposure, which may confound results.

• Here we describe a methodological approach to produce worm-shaped food packages, that is a practical diet, of controlled diameter and length (in the millimeter range), which allows to prepare food rations with a weight in the order of a few milligrams and to adjust the food rations to the individual body wet weight of small experimental fish with high accuracy (±0.5 mg) without the need for weighing/proportioning the feed using a scale.
• The method can be used to prepare test feed with *internally incorporated* particulate contaminants, such as manufactured nanomaterials and nano- and microplastics, to assess the latter's dietary uptake, bioaccumulation and associated toxicity in fish. We described two protocol variations: One using dry starting material, such as feed flakes, and one using liquid starting material, such as worm homogenate.
• The method has been developed for academic research environments with no access to specialized equipment for test feed preparation, and uses utensils and inexpensive plastic ware belonging to the standard inventory of ecotoxicological research laboratories.

## Graphical abstract

**Figure:**
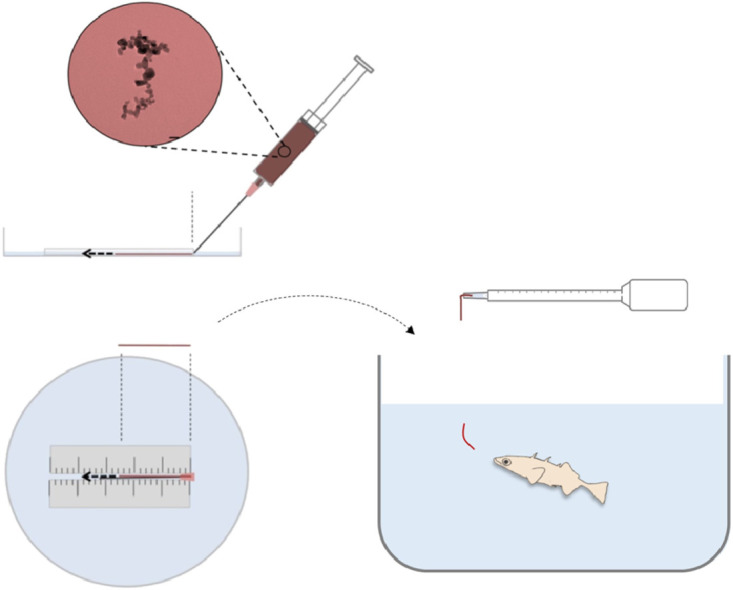

Specifications table

**Table:** 

Subject Area:	Environmental Science
More specific subject area:	Ecotoxicology
Method name:	Controlled dietary exposure of small experimental fish to nano- and microparticulate contaminants
Name and reference of original method:	Not applicable.
Resource availability:	Not applicable

## Method details

In the following, we describe two variations of a methodological approach we have developed to prepare test feed for controlled dietary exposure studies to nano- and microparticulate contaminants in small experimental fish. The first variation, denoted protocol A, describes the preparation of a worm-shaped practical diet (food packages) from dry starting material, such as commercial feed flakes or worm meal (e.g., ground freeze-dried worms). It can also be used to prepare small feed pellets. This variation has been successfully used to study dietary uptake and effects of titanium dioxide (TiO_2_) nanoparticles (NPs) in brown trout (*Salmo trutta*) fry (1–2 g body wet weight) [1]. The second variation (denoted protocol B) describes the preparation of a worm-shaped practical diet (food packages) from liquid starting material, specifically tissue homogenate of natural prey organisms of small fish (e.g., blended mosquito larvae or aquatic oligochaete worms). This protocol has been successfully used to examine dietary uptake and effects of copper-bearing NPs in three-spined stickleback (*Gasterosteus aculeatus*) (< 1 g) [2,3].

**Fig. 1:**
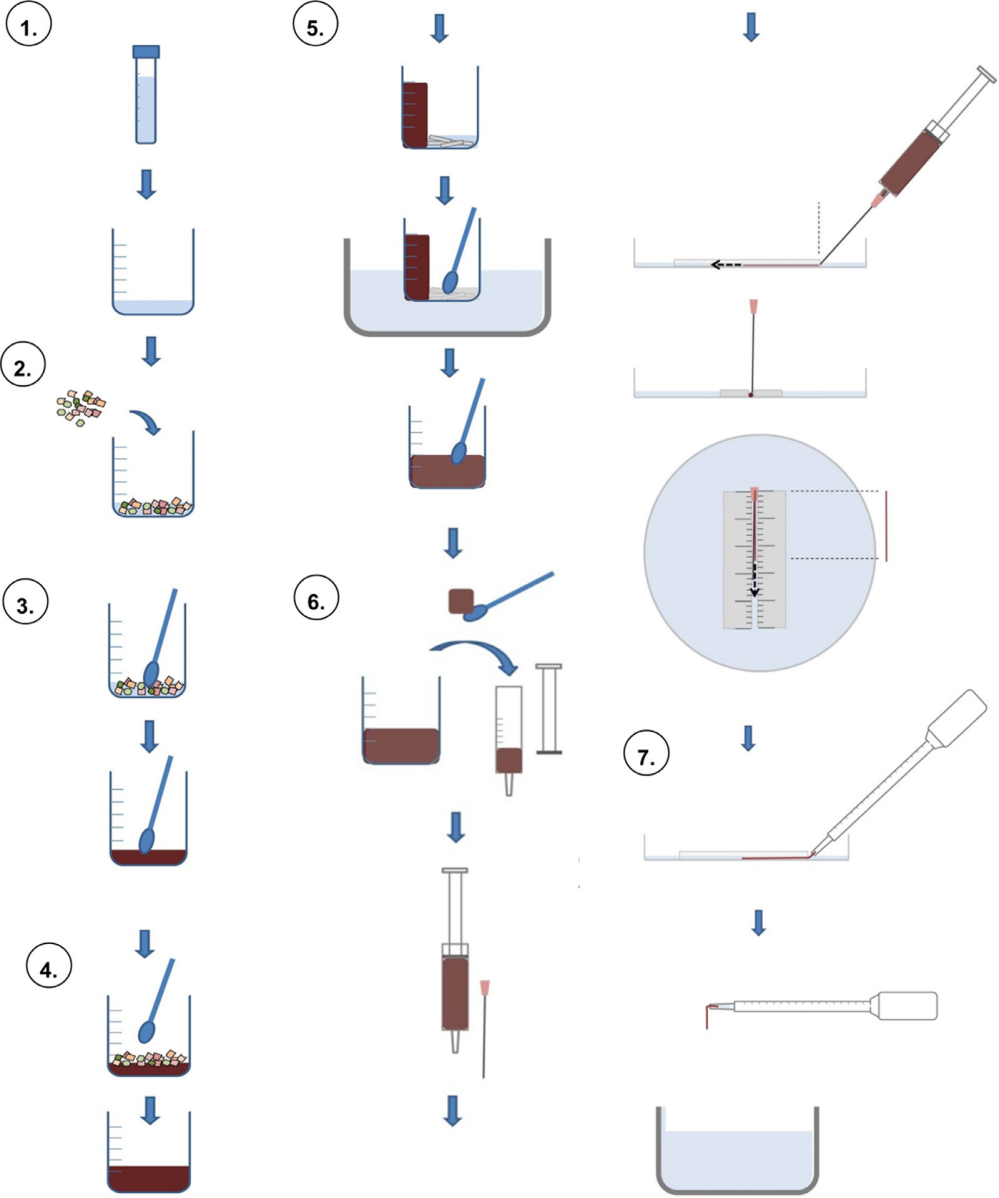
Feed preparation from dry starting material. Step 1: Preparation and characterization of the NP stock suspension. Step 2: Pre-sorption of the test substance(s) onto commercial fish food flakes. Step 3: Maceration and homogenization. Step 4: Up-scaling to the required food mass and dilution to the target concentration. Step 5: Optimization of the consistency and viscoelastic properties of the test food. Step 6: Proportionating the test food into rations of defined shape, weight and size. Step 7: Food manipulation and feeding.

NOTE: Depending on the research field and question and addressed, at this point of the protocol a second test compound (e.g., a suspected environmental co-contaminant, nutrient supplement, or veterinary pharmaceutical) can be spiked into the NP suspension if desired. Incubate the NP suspension with the second test compound under continuous stirring to allow both substances to interact, reach chemical equilibrium, and any organic solvent (if used) to evaporate.

A. **Feed preparation from dry starting material**
1. **Preparation and characterization of the NP stock suspension** (Fig. 1, step 1)
  1.1 Prepare a stable NP stock suspension in aqueous medium (e.g., in Milli-Q water using ultrasonication as described in [4]).
  1.2 Take aliquots of the NP suspension for characterization purposes (e.g., for determination of NP/alggomerate size, shape, hydrodynamic size distribution, colloidal stability etc. using transmission electron microscopy, dynamic light scattering or other appropriate analytical techniques) and for validation of the suspension's concentration (e.g., using ICP-MS for metal-bearing particles or photo-/fluorospectrometric analysis for colored/fluorescent particles/nanomaterials) [2], [3], [4], [5].
  1.3 Add a defined volume (in this example, 2.475 ml) of the NP stock suspension (or characterized dilutions thereof) to a clean glass beaker (recommended dimension: 100 ml capacity, 50 mm diameter, 70 mm height). Fill another beaker with the same volume of Milli-Q water for the preparation of the control diet.

2. **Pre-sorption of the test substance(s) onto commercial fish food flakes** (Fig. 1, step 2)
  2.1 Add commercially available fish feed flakes (dry) to the beaker at a weight that corresponds to the volume of the previously added aqueous solution/NP suspension (in this example, 2.5 g)
  2.2 Leave the feed flakes soaking in the solution for at least 5 min to allow the NP (and other associated test substances) to sorb to their surface.
3. **Maceration and homogenization** (Fig. 1, step 3)
  3.1 Macerate the soaked flakes using a stainless steel spoon until the content of the beaker glass has reached the consistency of a thick paste.
  3.2 Mix the food paste for at least 5 min to ensure homogenous distribution of the NP.
4. **Up-scaling to the required food mass and dilution to the target concentration** (Fig. 1, step 4)
  4.1 Step-wise add more feed flakes in small portions together with equal amounts of ultrapure water (e.g., 3 × 2.5 g flakes and 2.5 ml water) to the beaker and intermix it with the food paste.
  4.2 Make sure that each step is followed by a maceration and homogenization phase of at least 5 min as described under 3. (see above).
5. **Optimization of the consistency and viscoelastic properties of the test food** (Fig. 1, step 5)
  5.1 Stick the food paste to the wall of the glass beaker to create a free area at its bottom.
  5.2 For 20 g of food paste add 1 g of gelatin together with 5 ml of Milli-Q water to the beaker (both gelatin leafs cut into small pieces or gelatin powder can be used).

NOTE: In the study where this protocol was first used, ordinary food-grade gelatin as it is sold in food product markets was used (Dr Oetker Sverige AB, Sweden). However, we recommend to use gelatin produced for life science research in future studies, for example, gelatin tested according to European Pharmacopoeia (Ph. Eur.), which is available at established life science suppliers, to ensure consistency of quality across studies. We used such gelatin in protocol B (CAS: 9000–70–8, Ph.Eur., VWR international).

5.3 Soak the gelatin for at least 5 min in the added water and then dissolve it by placing the beaker in or over a warm water bath while stirring (a temperature of 40 °C was found to be sufficient).
5.4 Intermix the dissolved gelatin gradually with the food paste/dough.

NOTE: The mixing should be carried out for a time that is long enough to guarantee homogeneous distribution of the gelatin throughout the food paste (at least 5 min).

6. **Proportionating the test food into rations of defined shape, weight and size** (Fig. 1, step 6)
  6.1 Fill the test food into a disposable syringe (5 ml volume)
  6.2 Attach a microlance/hypodermic needle (e.g., 1.20 × 50 mm) to the adaptor of the syringe

NOTE: The microlance-equipped syringe will be used to produce worm-shaped food rations (food packages) of defined diameter and length. The length can be adjusted, but the maximum diameter is determined by the size of the aperture of the microlance/hypodermic needle. Food packages with diameters smaller than 1.20 mm can be produced by equipping the syringes with a different microlance/hypodermic needle, thicker food packages can be produced by extruding the food paste directly through the aperture of syringe (i.e., without microlance attached).

NOTE: Choose syringes that are free of latex, rubber, silicone oil, styrene, bis(2-ethylhexyl) phthalate (DEHP) or any other type of lubricant.

NOTE: At this stage the protocol can be paused. The food paste-filled syringes can be stored at 4 °C if they are used for preparation of food rations during the same week or at −20 °C if to be used at a later time point.

6.3 Prepare petri-dishes for production of worm-mimics (can also be prepared beforehand):

Cut two cable ties to a length of 4 cm and glue them on the (inner) surface of a tissue culture dish (60 mm diameter) using silicone aquarium sealant (free of any toxic additives such as fungicides). The space between the cable ties should be ~2 mm.

6.3.1 Cut a graph millimeter paper to the shape and size of the used tissue culture dish and stick/glue it to the bottom of it (i.e., the outer surface) with the graticule facing upwards (see Video 1).
6.3.2 Add ice-cold water (DI or Milli-Q) to the tissue culture dish until the water slightly covers the cable tie guide track
  NOTE: Instead of the tissue culture dish an ordinary plastic petry dish can be used. However, the tissue culture-treated surface, which is more hydrophilic, facilitates wetting of its surface and lower water volumes can be used).

6.4 Position the tip of the microlance/hypodermic needle at the opening of the scaled guide track (position A) and gently exert pressure, that is, push the plunger into the barrel of the syringe. (Fig. 1, step 6; Video 1)
6.5 Monitor how the “food worm” moves towards the other end of the scaled guide track (position B)
6.6 Cut the “food worm” at position A using a stainless steel scalpel (or razer blade) when the desired length in mm is reached. (Fig. 1, step 6; Video 1)

NOTE: The appropriate length depends on the density of the test feed and the amount of feed that shall be administrated per ration (If the food is prepared as in the example protocol, 10 mm of a 1.20 mm diameter “food worm” will weigh ~7.6 mg. For a fish weighing 1 g, which shall receive a daily ration of 2% of its body weight, that is 20 mg, the length of the “food worm” needs to be ~26 mm).

NOTE: The rations for each fish can be produced on a daily basis. The food paste-filled syringes can be stored at 4 °C if they will be used for preparation of food rations during the same week or at −20 °C if they will be used at a later time point.

7. **Food manipulation and feeding** (Fig. 1, step 7; Video 1)
  7.1 Use a disposable Pasteur pipette to remove any potential debris/NP released from the cut end of the worm-like food packages and carefully replace the water in the dish with fresh, clean ice-cold water.
  7.2 Gently collect the worm-shaped food package using a disposable Pasteur pipette and transfer it to the aquarium containing the experimental fish

NOTE: The prepared food rations can also be dried (at room temperature or better at 40 °C) to produce small feed pellets.

**Fig. 3:**
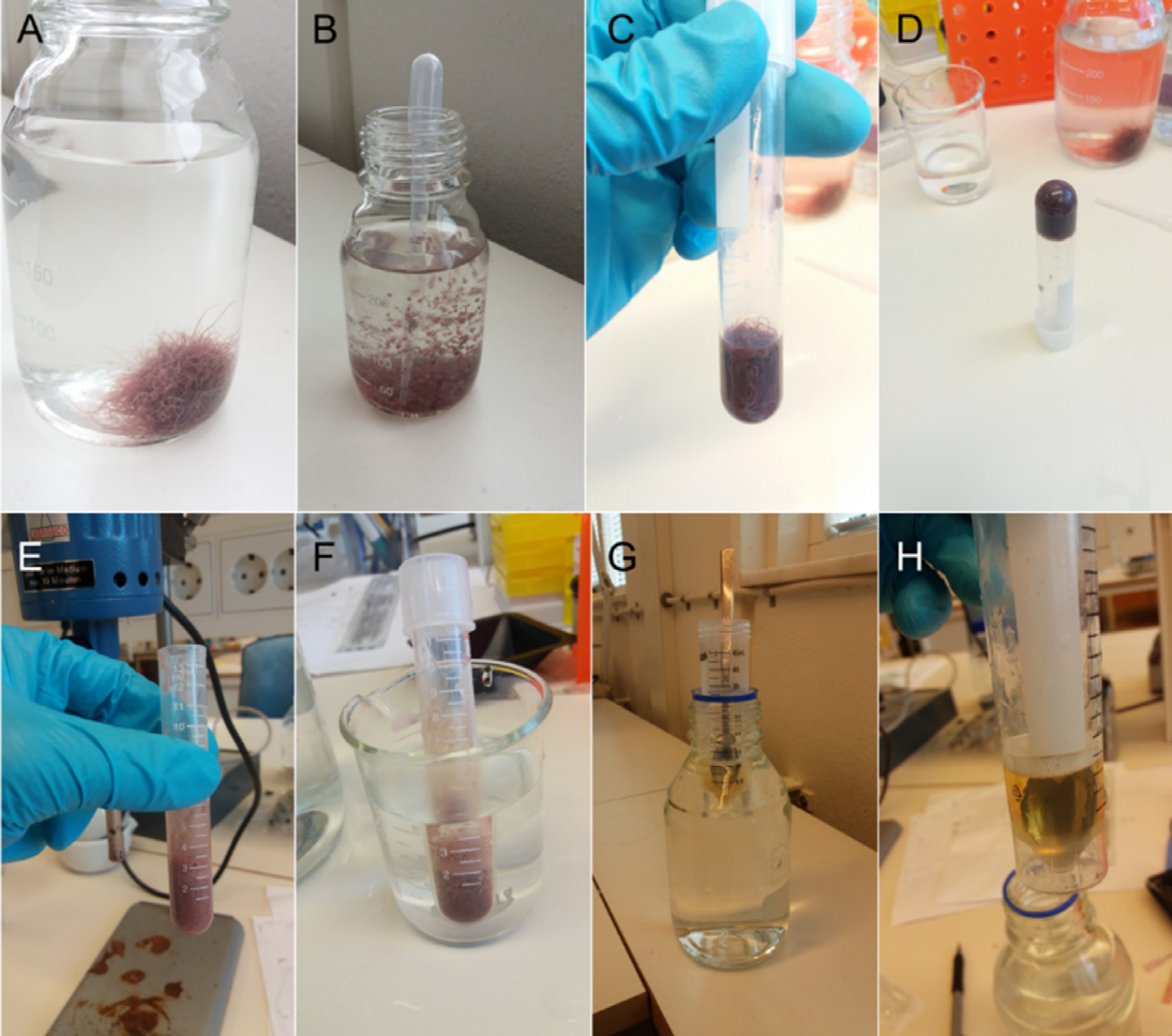
Photographs of steps 1–3 of protocol B. Collection of live worms (A-D). Preparation of worm homogenate (E, F). Preparation of gelatine solution (G, H).
    NOTE: *T. tubifex* worms entangled to balls can be easily individualized by repeatedly pushing water with the Pasteur pipette onto the worms.
    NOTE: Instead of worms from an in-house culture, live or frozen worms from a commercial supplier (e.g., red mosquito larvae) can be used to prepare the homogenate (see 2.).
  1.3 Aspirate all excess water using a micropipette until the tube can be inverted without losing its content. Weigh the tube, and subtract the weight of the empty tube (see 1.2) to determine the net weight of the worms (a photograph is shown Fig. 3).

B. **Feed preparation from liquid starting material**
1. **Culture and collection of live worms** (Fig. 2, step 1)
  1.1 Keep worms (in this example, *Tubifex tubifex*) in a sediment-free culture vessel in appropriate medium (e.g., artificial freshwater) for ~2 weeks before starting with the test feed preparation to ensure that worms have underwent depuration.
  1.2 Afterwards, collect the worms using a disposable Pasteur pipette and transfer them to a 13–15 ml round base-reaction tube (a photograph is shown Fig. 3). Determine the weight of the reaction tube beforehand.

**Fig. 2:**
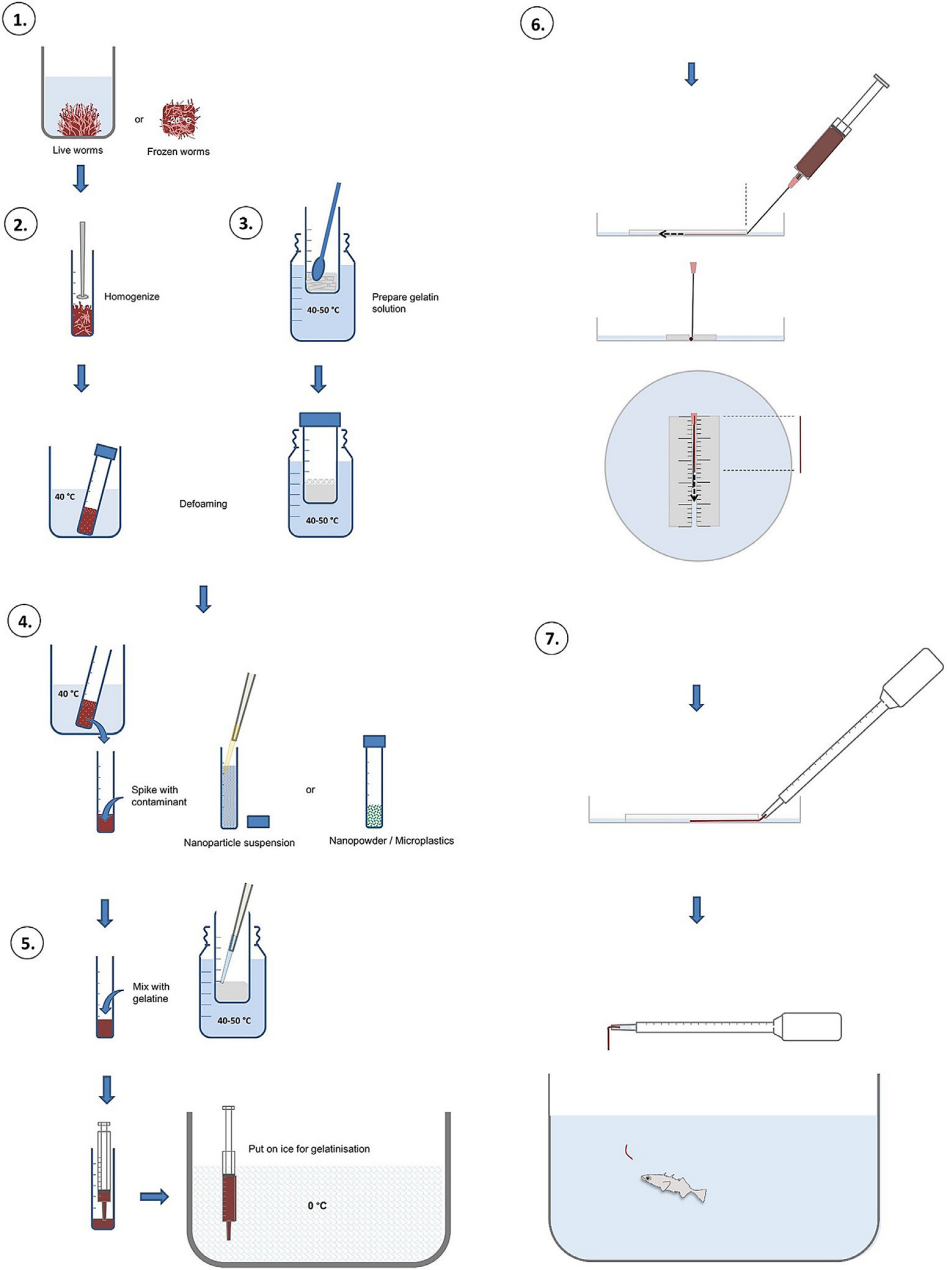
Food preparation from liquid starting material. Step 1. Culture and collection of live worms. Step 2. Preparation of worm homogenate. Step 3. Preparation of gelatine solution. Step 4. Incorporation and of the NP. Step 5. Reconstitution of a solid consistency. Step 6. Proportionating the test food into rations of defined shape, weight and size. Step 7. Food manipulation and feeding.
2. **Preparation of worm homogenate** (Fig. 2, step 2)
  2.1 Homogenize the worms using a rotor-stator type tissue homogenizer (here from Labassco AB; a photograph of the tissue homogenizer is shown in Fig. 3). For small volumes of approximately 5 ml a homogenization time of ~30 s is sufficient.
  2.2 Spike the homogenate with red food color (≤ 5% v/v) to reconstitute a “natural” color, which is lost due to the oxidation of hemoglobin.
    NOTE: It was observed that food packages with red color/higher contrast are recognized and consumed with higher efficiency than food packages without the colorant. This may however be specific for the model fish species used in our study, that is three-spined stickleback. When working with other fish species and or when using red mosquito larvae as starting material to prepare the homogenate it may not be necessary to add red food color.
  2.3 Place the tube with the foamy homogenate in a beaker containing tepid tap water (~30–40 °C) and wait until all air bubbles have risen to the surface and the foam has largely disappeared (a photograph is shown Fig. 3). In the meanwhile proceed with step 3.
3. **Preparation of gelatine solution** (Fig. 2, step 3;)
  3.1 Prepare a 0.3 g/ml gelatine solution by soaking an appropriate amount of gelatine powder (CAS: 9000–70–8, Ph.Eur., VWR international) in ultrapure water (DI or Milli-Q) and mixing it well with a stainless steel spoon while holding the reaction tube (in this example a 50 ml Falcon tube) into a warm water bath (40–50 °C) (a photograph is shown Fig. 3).
    NOTE: When working with small volumes of worm homogenate all steps can be carried out relatively quickly and lower gelatine concentrations (e.g., 0.2 g/ml) can be used for the feed preparation. When working with larger volumes of worm homogenate steps 4 and 5 tend to take longer and part of the gelatine appears to be degraded by hydrolytic enzymes (proteases) in the homogenate. Therefore, to begin with it is recommended to work with slightly higher gelatine concentrations (0.3 g/ml).
  3.2 Leave the liquid gelatine solution in a warm water bath until all bubbles have risen to the surface and the foam has largely disappeared. Then take an aliquot of a defined volume and measure its weight to determine the density of the gelatine solution (a photograph is shown Fig. 3).
4. **Incorporation of the NP** (Fig. 2, step 4)
  4.1 Calculate the volume of NP stock suspension that need to be spiked into the homogenate in order to obtain the desired concentration in the test feed (µg NP per g wet weight diet) taking into account the previously determined density of the worm homogenate and gelatine solution, and their mixing ratio (1:1; see step 5.1).
  4.2 Transfer the bubble-free part of the worm homogenate to a new reaction tube and add a defined volume of NP stock suspension to it. Then, gently pipette up and down and/or stir well until the NP is homogenously dispersed. Do *not* vortex.
    NOTE: The volume of the NP stock suspension added to the homogenate should be as small as possible in order not to dilute the homogenate, but large enough to still enable accurate pipetting. Therefore one should give particular consideration to prepare a NP stock suspension with an adequate concentration.

5. **Reconstitution of a solid consistency** (Fig. 2, step 5)
  5.1 Mix the NP-spiked homogenate and the gelatine solution in a 1:1 (v/v) ratio.
    NOTE: Make sure that the gelatine solution added to the homogenate is free of any air bubbles. To achieve this, prepare a large volume of gelatine solution, keep it incubated in a warm water bath until all bubbles have risen to the surface and then use and aliquot from the bottom of the tube.
    NOTE: Make sure that the homogenate has a similar temperature (~40 °C) as the gelatine solution before intermixing them. Otherwise (for example, when the homogenate would be taken directly from a fridge) because the gelatine will form lumps.
  5.2 Draw up the (differently) spiked homogenates into disposable syringes (in our study we used 1 ml) equipped with a microlance/hypodermic needle (e.g. 1.20 × 50 mm) and stick them immediately into an ice-filled box to initiate gelatinisation. After 30–60 min, transfer the syringes to a fridge, seal the tip with parafilm, and store at 4 °C overnight or until further use.
    NOTE: The microlance-equipped syringe will be used to produce worm-shaped food rations of defined diameter and length. The length can be adjusted, but the maximum diameter is determined by the size of the aperture of the syringe / microlance, that is, food “worms” with diameters smaller than 1.20 mm can be produced by equipping the syringes with a different variation of microlance.
    NOTE: Choose syringes that are free of latex, ruber, silicone oil, styrene, bis(2-ethylhexyl) phthalate or any other type of lubricant.

6. **Proportionating the test food into rations of defined shape, weight and size** (Fig. 2, step 6; Video 1)
  6.1 Prepare scaled tissue-culture dish with cable tie-guide track as described under A. 6.3.
  6.2 Take the syringes from the fridge and stick them into ice (with the protective cap removed).
  6.3 Fill the tissue-culture dish with ice-cold water, and prepare “food worms” of a length corresponding to the desired dietary exposure doses. The procedure is described under A. 6.4.
7. **Food manipulation and feeding** (Fig. 2, step 7; Video 1)
  7.1 Use a disposable Pasteur pipette to remove any potential debris/NP released from the cut end of the worm-like food packages and carefully replace the water in the dish with fresh, clean ice-cold water.
  7.2 Gently collect the worm-shaped food package using a disposable Pasteur pipette and transfer it to the aquarium containing the experimental fish.

## Method validation

The protocol (variation 1) yields food ‘dough’ with viscoelastic properties that allow its easy manipulation and further processing into worm-like food packages with defined diameter, length and mass. Both thick and thin food worms can be produced. The length of the worm-like food packages can be adjusted with high accuracy. The maximally expected deviation from the nominal length is 0.5 mm. For test food with a density comparable to that in our study (~0.7 g/cm^3^) this corresponds to < 0.5 mg. The weight of a worm-like food packages of 10 mm length and 1.2 mm diameter prepared via the above protocol is ~7.7 mg. The density, and hence weight of worm-like food packages prepared using a starting material (food flakes, homogenate etc.) with different composition and/or moisture content than that used in our study may slightly differ. It will also depend on the type and amount of nanomaterial incorporated into the food packages. However, we expect the differences to be rather small. In the two case studies with TiO_2_ NP and CuO NPs, the differences between the weight of food containing different amounts/concentrations of nanomaterial were negligible. Furthermore, the weight between food packages prepared from dry food (flakes) and liquid (tubifex homogenate) starting materials was similar. Both flake-based and homogenate-based food packages maintain their structural integrity during up to 8 h in cold water (highest temperature tested: 14 °C). Some swelling may occur, but food packages are usually quickly consumed (see Video 1). Food packages prepared from flakes were efficiently consumed by brown trout fry (body wet weight ~1–2 g). Food packages prepared from tubifex homogenate were consumed within few seconds by three-spined stickleback (body wet weight ~1–2 g) (Video 1). No nanomaterial-dependent food avoidance (e.g., as a consequence olfactory sensing of the contaminant) was observed for either of the food package types and metal oxide NPs (TiO_2_, CuO) and concentrations (maximum concentrations tested: 1 mg/g TiO_2_/wwt food, and 50 µg/g Cu/wwt food). Which of the two presented protocols is more appropriate for a specific study may depend on various factors including the specific research question, model fish species, and type of material (particulate contaminant). For example, for studies investigating trophic transfer of particulate contaminants from aquatic invertebrates to fish, protocol variation B may be the method of choice, as it does not only allow the preparation of *spiked* test food, but also the preparation of test food using tissue homogenate of invertebrates that were *previously* exposed to the test substance (e.g., oligochaete worms exposed to sediment containing environmentally relevant concentrations of nanomaterial [6]). The comparison of both food types can help to determine the influence of biological incorporation (biological transformation) of the test substance by the invertebrate on its dietary uptake and accumulation by the predator (fish). Furthermore, it needs to be taken into consideration that the attractability of food prepared according to protocol A and food prepared according to protocol B, which differ in their sensorial properties (chemical and physical characteristics), may depend on the food preferences/gustatory system of the model fish species used. For example, we observed that food prepared *via* protocol A was efficiently consumed by trouts but not accepted by sticklebacks. Another factor that may be important to consider when deciding which protocol variation may be the most suitable for a study is the physico-chemical properties of the particulate contaminant. For example, when working with nanomaterials/particles that readily dissolve in aqueous solutions, like it may be the case for some non-surface modified metal NPs, protocol variation A, which uses a dry starting material (flakes, flour), may be more appropriate (or a modification of it where the nanopowder is directly added to the food dough, instead of first dispersed in aqueous solution). Protocol variation A may also be more suitable for particles that have a high buoyancy, such as low-density microplastics, as these, when spiked into the low viscous tissue homogenate (protocol variation B), might rise to the top of the homogenate-filled syringe resulting in uneven distribution of particle throughout the food, and hence variability in exposure dose. This risk is minimized/eliminated using protocol variation A, where the food has a dough-like consistency. Protocol variation B, on the contrary, is recommendable, when information on the particles’ morphological characteristics *in* the food (e.g., size, shape, agglomeration state) is needed. Thus, we were able to fix and resin-embed the food prepared according to protocol B, and image electron-dense particles (CuO NPs) in ultrathin sections of the food using transmission electron microscopy [2].

## Appendix Supplementary materials

Image, video 1
